# Enzyme-free Passage of Human Pluripotent Stem Cells by Controlling Divalent Cations

**DOI:** 10.1038/srep04646

**Published:** 2014-04-11

**Authors:** Kiyoshi Ohnuma, Ayaka Fujiki, Kana Yanagihara, Saoko Tachikawa, Yohei Hayashi, Yuzuru Ito, Yasuko Onuma, Techuan Chan, Tatsuo Michiue, Miho K. Furue, Makoto Asashima

**Affiliations:** 1 Top Runner Incubation Center for Academia-Industry Fusion, Nagaoka University of Technology, 1603-1 Kamitomioka, Nagaoka, Niigata 940-2188, Japan; 2 Department of Life Sciences (Biology), Graduate School of Arts and Sciences, The University of Tokyo, 3-8-1 Komaba, Meguro, Tokyo 153-8902 Japan; 3 Laboratory of Stem Cell Cultures, Department of Disease Bioresources Research, National Institute of Biomedical Innovation, 7-6-8, Saito-Asagi, Ibaraki, Osaka 567-0085, Japan; 4 Research Center for Stem Cell Engineering, National Institute of Advanced Industrial Science and Technology (AIST), Tsukuba Central 4, 1-1-1 Higashi, Tsukuba, 5 Ibaraki 305-8562, Japan

## Abstract

Enzymes used for passaging human pluripotent stem cells (hPSCs) digest cell surface proteins, resulting in cell damage. Moreover, cell dissociation using divalent cation-free solutions causes apoptosis. Here we report that Mg^2+^ and Ca^2+^ control cell-fibronectin and cell-cell binding of hPSCs, respectively, under feeder- and serum-free culture conditions without enzyme. The hPSCs were detached from fibronectin-, vitronectin- or laminin-coated dishes in low concentrations of Mg^2+^ and remained as large colonies in high concentrations of Ca^2+^. Using enzyme-free solutions containing Ca^2+^ without Mg^2+^, we successfully passaged hPSCs as large cell clumps that showed less damage than cells passaged using a divalent cation-free solution or dispase. Under the same conditions, the undifferentiated and early-differentiated cells could also be harvested as a cell sheet without being split off. Our enzyme-free passage of hPSCs under a serum- and feeder-free culture condition reduces cell damage and facilitates easier and safer cultures of hPSCs.

Human pluripotent stem cells (hPSCs), including human embryonic stem cells (hESCs) and human induced pluripotent cells (hiPSCs), have increased the possible applications of stem cell research in biology and medicine^1^,^2^,^3^. Since dissociating hPSCs into single cells using divalent cation-free solution causes cell damage and death by apoptosis^4^,^5^,^6^,^7^,^8^, hPSC passaging usually entails dissociating the cell colonies into large cell clumps using enzymes in a divalent cation-containing solution (Table 1). However, these enzymes may also induce cell damage by digesting cell-surface proteins^5^,^8^.

To achieve enzyme-free and less damaging passage of hPSCs, we focused on the roles of Ca^2+^ and Mg^2+^ in cell-cell and cell-fibronectin binding. Physiological concentrations of Ca^2+^ regulate cell-cell binding of hPSCs mediated by E-cadherin^2^,^7^,^9^,^10^. On the other hand, physiological concentrations of Mg^2+^ are required for optimal, tight binding between cells and fibronectin, part of the extracellular coating matrix of hPSCs^11^,^12^,^13^,^14^,^15^. We therefore hypothesized that solution containing physiological concentration of Ca^2+^, but no Mg^2+^, could be used to passage hPSCs cultured on fibronectin-coated dishes as large cell clumps without the need for enzyme-based cell dissociation. We tested this hypothesis using our serum- and feeder-free culture medium (ESF9a) in fibronectin-coated dishes^14^,^16^,^17^, allowing us to examine hPSC attachments without masking by undefined adherent factors derived from the serum and feeder cells.

## Results

### Dose-dependent effects of Mg^2+^ and Ca^2+^ on cell-fibronectin and cell-cell binding

**Figure 1:**
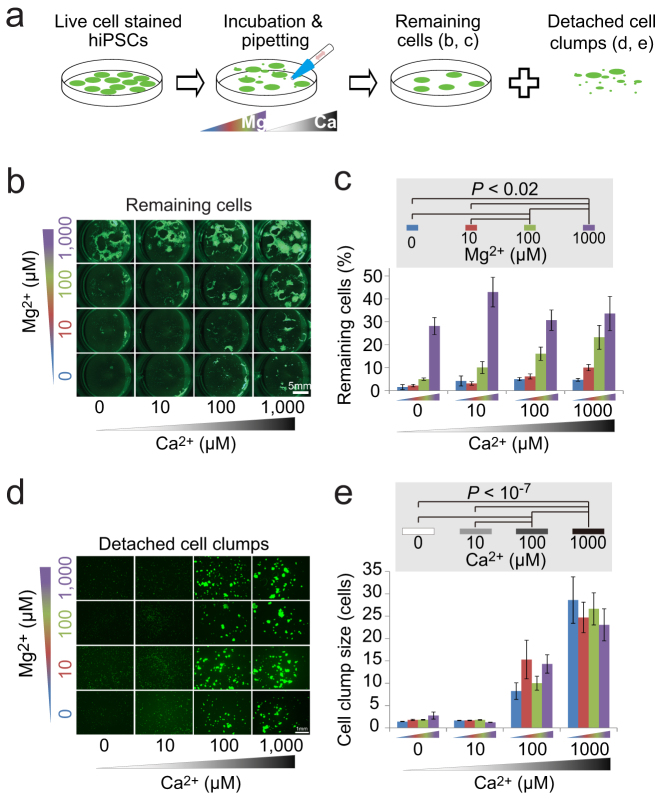
Dose-dependent effects of Mg^2+^ and Ca^2+^ on cell-fibronectin and cell-cell binding. (a): Schematics of experiments. The illustrations were drawn by KO. (b–e): Fluorescent imaging (live-cell dye, Calcein-AM) of the hiPSCs (253G1) remaining on the fibronectin-coated 24-well plate (b) and the cells detached from the plate (d) after incubation and then trituration using a 1-ml pipette tip in PBS containing 0–1000 μM Ca^2+^ and Mg^2+^. (c): Remaining-cell ratios equal the area of remaining cells divided by the area of the cells before incubation and trituration. (d): Mean cell clump size detached from the plates. Two-way ANOVA revealed no interaction effect between Ca^2+^ and Mg^2+^ concentration ((c): *P* = 0.066, mean ± SE, n = 5, (e): *P* = 0.47, mean ± SE, n = 5 experiments × 200 cells). Post-hoc Tukey's multiple comparison revealed significant differences in remaining-cell ratio between different Mg^2+^ concentrations (the same Ca^2+^ data were put together to derive the numbers and bars in (c)) and in cell clump size between different Ca^2+^ concentrations (the same Ca^2+^ data were put together to derive the numbers and bars in (e)). Scale bars are 5 mm (b), 1 mm (d).

The hiPSCs 253G1^18^ and 201B7^2^ were incubated in phosphate-buffered saline (PBS) containing various concentrations (0, 10, 100, 1000 μM) of Mg^2+^ and Ca^2+^ and then triturated to detach cells from the fibronectin-coated plates and dissociate them into cell clumps (Fig. 1a). The number of cells remaining on the dishes decreased with decreasing Mg^2+^ concentration, although the sizes of the detached hPSCs clumps increased with increasing Ca^2+^ concentration (253G1: Fig. 1b–e, 201B7: Supplementary Fig. 1ab). These results suggest that the cell-fibronectin binding depended on Mg^2+^ concentration whereas cell-cell binding of hPSCs was dependent on Ca^2+^ concentration, and that these bindings could be independently controlled without enzyme.

### Passage of hPSCs with enzyme-free solution containing a physiological concentration of Ca^2+^ without Mg^2+^

We tested whether the buffer solutions with Ca^2+^ and without Mg^2+^ could be used to passage hPSCs. Large cell colonies of hESCs, HUES8^19^, and H9^1^ were detached from fibronectin-coated dishes and dissociated into large cell clumps by incubation in PBS containing 1 mM Ca^2+^ without Mg^2+^ (PBS^ca/−^) followed by pipetting (Supplementary Fig. 2a–g). These detached cell clumps were then plated into fibronectin-coated dishes and reattached as typical hPSC flat colonies on the next day (Supplementary Fig. 2h–m). In addition, hiPSCs cultured on vitronectin and laminin, which are also used as a coating matrix for culturing hPSCs (Table 1), can be detached from the culture dishes by PBS^ca/−^ (Supplementary Fig. 3). These results suggested that enzyme-free solution containing physiological concentration of Ca^2+^, but no Mg^2+^, could be useful for passaging hPSCs as large cell clumps.

### Effects of dissociation and enzymatic digestion

We compared our enzyme-free passage method to both dissociation passaging in a divalent cation-free solution and enzymatic digestion passaging.

**Figure 2:**
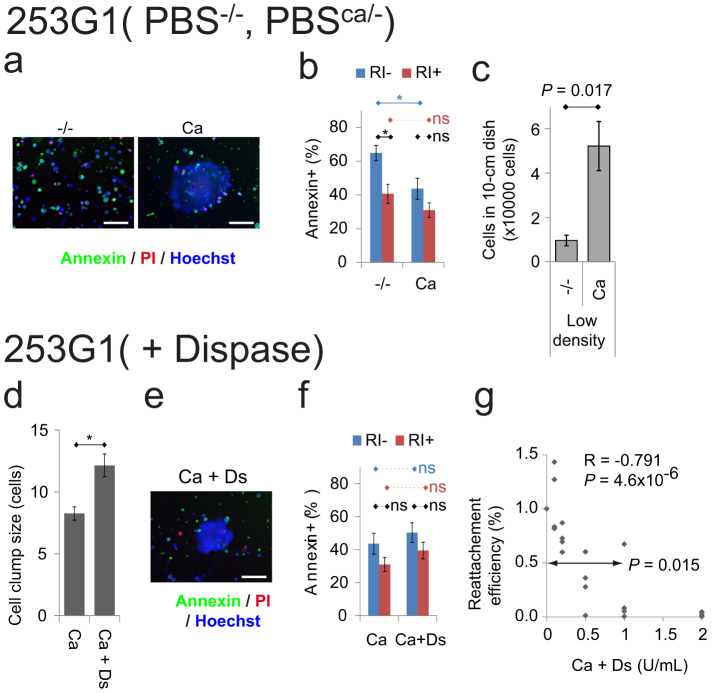
Effects of dissociation without divalent cation and with dispase. (a–c): Comparison between the hiPSCs (253G1) detached and dissociated by PBS^−/−^ (−/−) and those by PBS^ca/−^ (Ca). (ab): Micrograph (a) and FCM analysis (b) of apoptosis marker annexin V-FITC after four hours floating culture (RI: with 5 μM ROCK inhibitor) following detachment and dissociation. The cells were stained by annexin V-FITC (green), propidium iodide (red: late apoptosis or necrosis marker), and Hoechst 33342 (blue: nuclei marker). (c): The number of cells remaining after 3 days culture in ESF9a on fibronectin-coated 10-cm dishes (the initial cell number was 1.2 × 10^5^ cells/10-cm dish, mean ± SE, n = 5, t-test). (d–g): Effects of dispase in PBS^ca/−^ for detaching and dissociating cells. (d): Mean cell clump size detached from the plates using PBS^ca/−^ (Ca) and 1 U/ml dispase in PBS^ca/−^ (Ca + Ds). (ef): Micrograph (e) and FCM analysis (f) of annexin V-FITC after four hours floating culture following detachment and dissociation by PBS^ca/−^ or 1 U/ml dispase in PBS^ca/−^. The staining were the same as (a). (g): Reattachment efficiency. The cells were digested with 0–2 U/ml dispase in PBS^ca/−^ and were plated with ESF9a medium including 5 μM ROCK inhibitor. The numbers of cells were estimated using calcein-AM 1 day after plating and normalized against the PBS^ca/−^ results (0 U/ml dispase). The mean value at 1 U/ml dispase was smaller than 1 (*P* < 0.015, n = 4, t-test). Pearson's correlation coefficient = −0.791, *P* = 4.6 × 10^−6^ t-test, from 4 independent experiments. Scale bars are 100 μm. (bdf): * *P* = 0.05, ns: not significant, n = 6, mean ± SE (bf) and * *P* = 0.01, ns: not significant, n = 4 experiments × 200 cells, mean ± SE (d), Holm's multiple comparison tests (cf. Supplementary Fig 5).

Dissociating hPSCs into single cells and replating at low density causes cell damage and death by apoptosis^4^,^5^,^6^,^7^,^8^. Thus, we hypothesized that detaching and dissociating hPSCs into larger clumps using PBS^ca/−^ could suppress apoptosis and support higher survival rates than detaching and dissociating these cells into smaller clumps using PBS without Ca^2+^ and without Mg^2+^ (PBS^−/−^). To test this hypothesis, apoptosis was detected in the hiPSCs 253G1 and 201B7 using annexin V-FITC, which stains cell surface phosphatidylserine, four hours after detachment and dissociation in PBS^−/−^ and PBS^ca/−^ followed by floating culture in ESF9a solution. Fluorescence microscopy showed many annexin V-FITC-positive single cells dissociated by PBS^−/−^, whereas annexin V-FITC-negative cells predominated in the large cell clumps dissociated by PBS^−/ca^ (253G1: Fig. 2a, 201B7: Supplementary Fig. 4a). Quantitative analysis using flow cytometry (FCM) revealed that fewer annexin V-FITC-positive cells were detached and dissociated by PBS^ca/−^ than by PBS^−/−^, and that addition of a ROCK inhibitor (RI) abolished these differences (253G1: Fig. 2b, 201B7: Supplementary Fig. 4b). RI blocks the dissociation-induced apoptosis of hPSCs^6^,^7^. To measure cell survival, hPSCs were detached and dissociated in PBS^ca/−^ or PBS^−/−^, plated at low density (2 × 10^3^ cells/cm^2^), and then cultured for 3 days. The numbers of hiPSCs passaged in PBS^ca/−^ were higher than those passaged in PBS^−/−^ (253G1: Fig. 2c, 201B7 & Tic: Supplementary Fig. 4c), suggesting that adding physiological concentration of Ca^2+^ to the dissociation solution increases cell survival rates by decreasing dissociation-induced apoptosis.

It is also known that enzymatic digestion damages hPSCs^5^,^8^. We first used dispase, an enzyme often used to passage hPSCs under serum- and feeder-free conditions (Table 1)^20^. Because we routinely use 0.025–0.6 U/ml dispase (0.05–300 mg/ml), depending on the enzyme activity and on storage conditions^14^, excess dispase (1 U/ml) was used to evaluate its damaging effect with the expectation that dispase dissociation of cell-cell binding would decrease the size of cell clumps, resulting in apoptosis. However, addition of 1 U/ml dispase in PBS^−/ca^ did not decrease hPSC clump size (253G1: Fig. 2d, 201B7: Supplementary Fig. 4d). Indeed, large clumps of annexin V-FITC-negative cells were also found when dispase was added to the PBS^ca/−^ (253G1: Fig. 2e, 201B7: Supplementary Fig. 4e), and quantitative analysis by FCM revealed that the relative percentages of annexin V-FITC-positive apoptotic cells were not changed by addition of dispase (253G1: Fig. 2f, 201B7: Supplementary Fig. 4f). The results were the same when 0.25% trypsin was added to PBS^ca/−^, despite trypsin having more potent protease activity than dispase (Supplementary Fig. 5a–c, e–g). These findings together suggested that adding proteolytic enzyme to the PBS^ca/−^ dose not decrease the cell clump size and thus does not increase dissociation-induced apoptosis. Our results are consistent with a previous report that Ca^2+^ protects against trypsinization of cell-cell binding^10^. Because Ca^2+^ did not affect the cell-fibronectin binding during dissociation (Fig. 1bc, Supplementary Fig 1a), we next measured the re-attachment ability of hiPSCs to fibronectin-coated surfaces. To do this, hPSCs were incubated with dispase in PBS^ca/−^, and replated in ESF9a medium with RI for counting the next day. The reattachment efficiency decreased with increasing concentrations of dispase, and the mean efficiency values at 1 U/ml dispase were smaller than those for PBS^ca/−^ alone (253G1: Fig. 2g, 201B7: Supplementary Fig. 4g); a similar result was attained when trypsin was added to PBS^ca/−^ (Supplementary Fig. 5dh). These results suggested that addition of enzyme damages cells by suppressing cell-fibronectin rebinding rather than by increasing apoptosis.

These results showed that enzyme-free solution containing a physiological concentration of Ca^2+^, without Mg^2+^, enables passaging of hPSCs with less cell damage than found using either divalent-free solution or Ca^2+^-containing solution with enzyme (dispase or trypsin).

### Long-term culture of hPSCs with enzyme-free passaging

Next, we tried long-term culturing of hPSCs under enzyme-, serum-, and feeder-free culture conditions. Two hiPSC lines, 253G1 and 201B7, were successfully cultured for more than 10 passages in ESF9a medium on fibronectin-coated dishes by using the solution with Ca^2+^ and without Mg^2+^ (ESF-Fb-EzF condition), with both cell types expressing self-renewal markers (Supplementary Fig. 6a–c, j–l). Immunocytochemistry of embryoid bodies derived from the two cell lines indicated that pluripotency was maintained (Supplementary Fig. 6dm). Unexpectedly, karyotype abnormalities were found not only under the ESF-Fb-EzF condition (Supplementary Fig. 6ir) but also in the sister cultures under the other conditions (Supplementary Fig. 6hopq), suggesting that the abnormalities were induced before enzyme-, serum-, and feeder-free culture.

**Figure 3:**
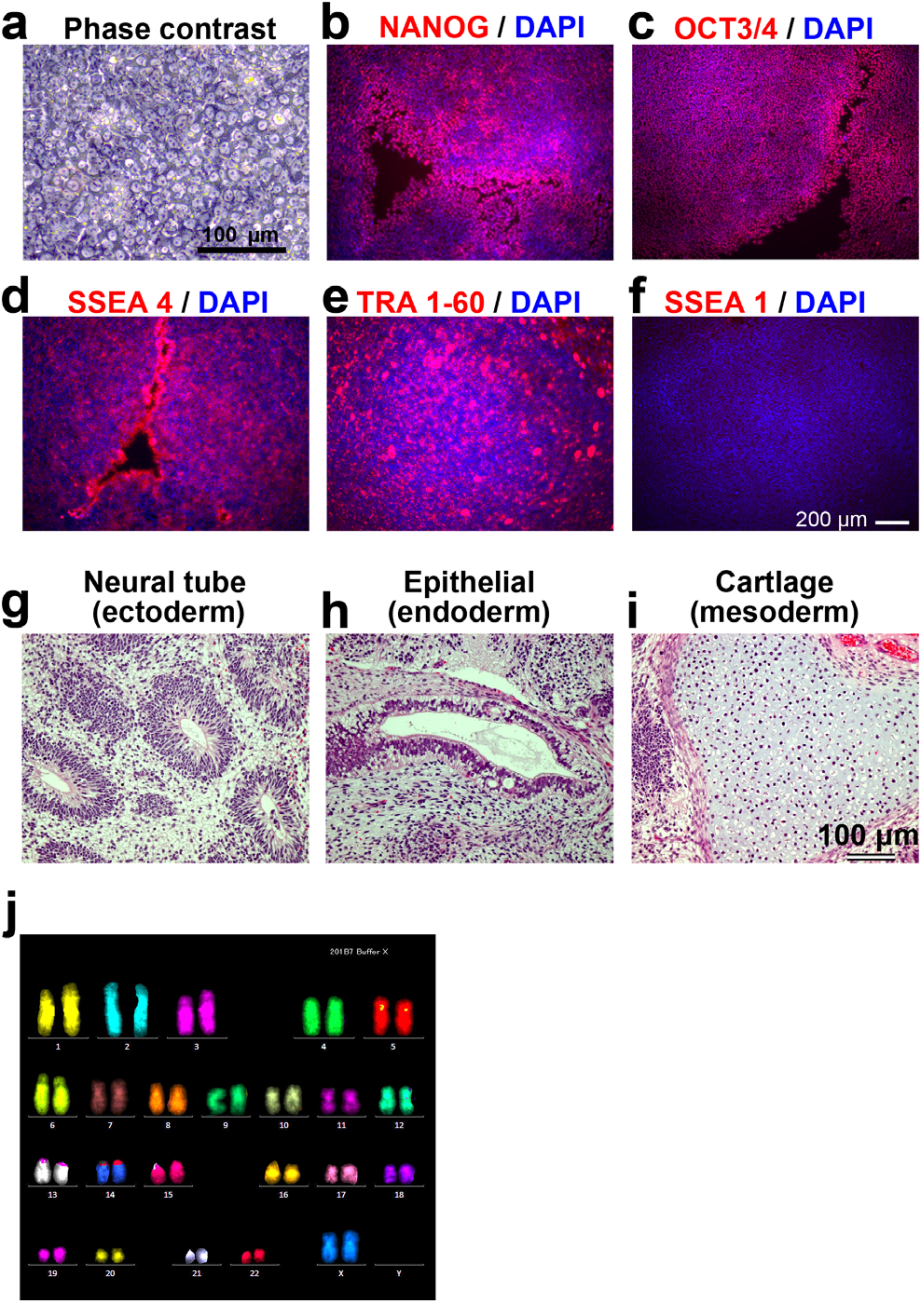
Passage solution with Ca^2+^ and without Mg^2+^ supports long-term culture and pluripotency of hPSCs. The hiPSC 201B7 were cultured for 15 passages under the ESF-Fb-EzF condition. (a): Phsecontrast micrograph. (b–f): Immunocytochemistry showed that the cells expressed self-renewal markers, NANOG ((b): red), OCT3/4 ((c): red), SSEA4 ((c): red) and TRA 1–60 ((e): red), but not an early differentiation marker, SSEA1 ((f): red). The nuclei were stained with DAPI (blue). (g–h): Histological analysis with HE staining demonstrated that hiPSC-derived teratoma contained derivatives of all three germ layers: neural tube ((g): ectoderm), epitherial ((h): endoderm), and cartridge ((i): mesoderm). (j): FISH karyotype analysis showed a normal karyotype (46XX). Scale bars are 100 μm (a, g–i) or 200 μm (b–f).

To confirm the karyotype normality, we newly performed long-term cultures using hiPSC 201B7 and Tic lines. The 201B7 cell line was pre-validated to ensure a normal karyotype, and then cultured under the ESE-Fb-EzF condition for more than 10 passages. The cells formed normal hiPSC colonies, which were tightly packed, and flat colonies consisting of cells with large nuclei and scant cytoplasm (Fig. 3a)^1^,^2^,^3^ and expressed four self-renewal markers, NANOG, OCT3/4, SSEA4 and TRA 1–60 but not an early differentiation marker, SSEA1 (Fig. 3b–f). The cells differentiated into derivatives of all three primary germ layers *in vivo* using teratoma formation (Fig. 3g–i). The cells showed a normal karyotype (Fig. 3j). Karyotype after long-term culture was also normal in another hiPSC line, Tic (Supplementary Fig. 7), confirming that karyotype remained stable during the enzyme-, serum-, and feeder-free culture. These results suggested that enzyme-free culture is a useful method for routine culturing of hPSCs.

### Cell sheet harvesting

**Figure 4:**
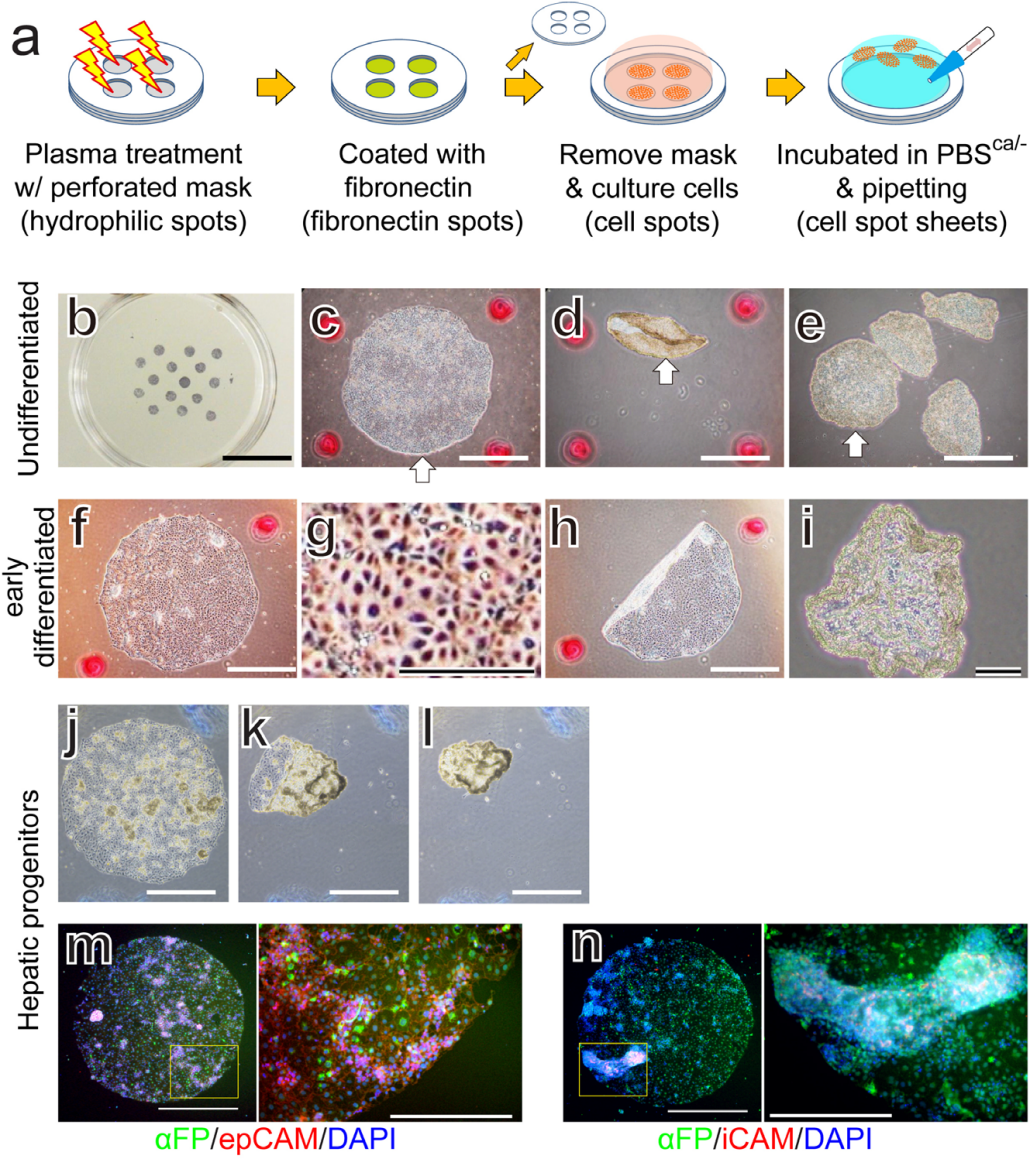
Cell sheet harvesting. (a): Schematics of spot sheet formation. The hiPSCs (253G1) were cultured in ESF9a medium (undifferentiated: (b–e)), ESF6 medium supplemented with BMP4 for two days (early differentiated: (f–i)). The hiPSC (Tic)-derived hepatic progenitors (j–n). The illustrations were drawn by KO. (b): ALP staining of the hiPSCs plated on 2-mm-diameter fibronectin spots. Phase-contrast micrographs before (c, f, g, j), during (h, k), and after (d, e, i, l) 15 min in PBS^ca/−^ followed by pipetting. (g) is a magnified image of part of (f). The white arrows indicate the same cell spot sheet (c, d, e). The red spots in (c–e, f, h, i) and blue irregular marks in (j–l) are position makers. (mn): Immunohistochemistry of hepatic progenitors using the early stages of liver development marker, α-fetoprotein (αFP: green), EpCAM (red), and iCAM (red). Nuclei were stained with DAPI (blue). The right panels are magnified images of the boxed areas in the left panels. Scale bars are 1 cm (b), 1 mm ((c–f, h, j–l), left of (mn)), and 400 μm ((g, i), right of (m,n)).

Finally, we tried cell sheet harvesting using our enzyme-free solution. Cell sheet harvesting using special equipment such as a temperature-responsive surface and magnet followed by transplantation is one of the most promising approaches for applying hPSCs to regenerative medicine^21^,^22^. However, in this study, simple incubation in PBS with Ca^2+^ followed by gentle pipetting enabled us to harvest the cells as 2-mm-diameter sheets without cells splitting off (Fig. 4a–e) and without specialized equipment. Similar results were obtained for early-differentiated cells induced by bone morphogenetic protein 4 (BMP4) (Fig. 4f–i) and for hiPSC-derived hepatic progenitors (Fig 4j–n), suggesting that the PBS with Ca^2+^ could be used to routinely and simply harvest cells as a large sheet without special equipment.

## Discussion

The present study showed that cell-fibronectin and cell-cell binding are controlled separately by Mg^2+^ and Ca^2+^, respectively, in hPSC cultures. Using enzyme-free solutions containing Ca^2+^ without Mg^2+^, we successfully passaged hPSCs cultured under serum- and feeder-free conditions as large cell clumps that showed less damage than those passaged in divalent cation-free solution or with dispase or trypsin. The cells were also harvested as a cell sheet without the need for splitting off.

The cell clumps dissociated by PBS^ca/−^ (1 mM Ca^2+^ and 0 mM Mg^2+^) and represented in Fig 2d were smaller than those represented in Fig 1e. The decreased cell clump size might be caused by the DNase added in all enzyme-related experiments (Fig 2d–g, and Supplementary Fig 4d–g and 5) to reduce the abundance of free-floating DNA fragments derived from damaged cells. Such addition of DNase might reduce cell-cell attachments arising from the free DNA fragments, and thereby also reduce cell clump size. However, even in the presence of DNase, the cell clumps dissociated by PBS^Ca/−^ without enzyme were significantly larger than those dissociated by PBS^−/−^ without enzyme (Supplementary Fig 5ae).

Addition of enzyme increased the sizes of cell clumps in three of the four conditions in the presence of calcium (Fig 2d, Supplementary Fig 4d and 5ae). A possible reason for this size increase tendency is enzymatic digestion of some cell-fibronectin attachment that enabled cell colonies to detach more easily from the dish. Consequently, large colonies may be harvested intact with less splitting of cell-cell binding by pipetting.

**Figure 5:**
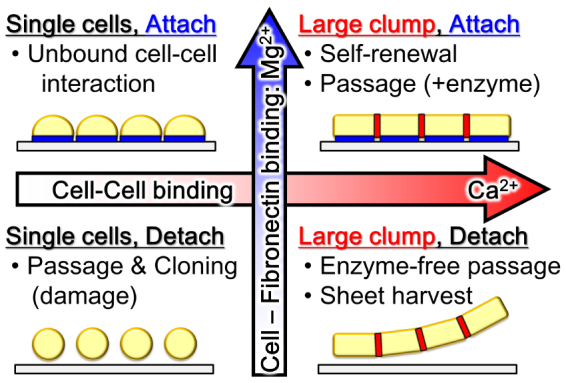
Schematics of the effects of Mg^2+^ and Ca^2+^ on hPSCs culture.

Commonly, hPSCs are passaged with enzyme and in medium containing physiological concentrations of Mg^2+^ and Ca^2+^ (Fig. 5 upper right)^1^,^2^. Single-cell culture methods such as clonal isolation are achieved by dissociating cells in solutions containing low Mg^2+^ and Ca^2+^ concentrations (Fig. 5 lower left)^4^,^6^,^8^. In the present study we showed that hPSC cell-cell binding can be disrupted with less cell detachment from the dish surface in a solution containing high Mg^2+^, but low Ca^2+^ concentrations (Fig. 5 upper left), and that large cell clumps and sheets can then be harvested by dissociating in low Mg^2+^ and high Ca^2+^ solution (Fig. 5 lower right).

The serum-, feeder-, and enzyme-free composition described herein could provide practical culture methods for controlling hiPSCs physical interactions and thus enable further studies into the effects of such interactions and of endogenous and exogenous factors on cells, with the added benefit of eliminating instability caused by lot differences in enzyme. Moreover, such defined culture conditions could facilitate a stable and safe source of hiPSCs for potential clinical applications.

## Methods

### hPSCs culture

The hESC HUES8^19^, H9 (WA09)^1^, KhES1, and KhES3^23^ lines were obtained from Harvard University (Cambridge, MA, USA), from WiCell Research Institute (Madison, WI, USA), or from Kyoto University (Kyoto, Japan). The hiPSC 201B7^2^ and 253G1^18^ lines were obtained from RIKEN BRC Cell Bank (Tsukuba, Ibaraki, Japan) through the National Bio-Resource Project for MEXT, Japan and the hiPSCs Tic^24^ line (JCRB13331), which was derived from fetal lung fibroblasts (MRC-5), was obtained from JCRB Cell Bank (Osaka, Japan). hPSCs were maintained in a KSR-based medium on mouse embryonic fibroblast (MEF) feeder cells, and subcultured using CTK medium (KSR-MEF-CTK condition), as described in Supplementary Methods. In all experiments, hPSCs maintained in KSR-based medium on MEFs were transferred into serum-free medium, hESF9a on fibronectin-coated dishes, and passaged at least once before assaying (Supplementary Methods). The culture dishes were coated with 2 μg/cm^2^ fibronectin from human plasma (063-05591; Wako) or from bovine plasma (F-1141; Sigma, St. Louis, MO, USA) in PBS for at least 30 min at 37°C, and then excess solution was removed. For subculturing, the cells were detached from the culture dish using 0.2–0.5 U/ml dispase (17105-041; Life Technologies, Grand island, NY, USA) in hESF9a medium and replated in hESF9a medium with 5 μM ROCK inhibitor (RI, Y-27632; Wako). The hESF9a medium (ESF-Fb-Dsp condition) was changed daily.

For long-term culture under enzyme-, serum- and feeder-free condition (ESF-Fb-EzF condition), the cells were passaged with enzyme-free passage solution containing divalent cation-free DMEM-F12 medium (Supplementary table 1) supplemented with the same factors found in ESF9a medium. For subculturing, the cells were rinsed twice with PBS^−/−^ and once with the enzyme-free passage solution, before being incubated in the same solution for more than 15 min at 37°C, and then triturated with a 1-ml micropipette tip. The cells were finally harvested by gentle centrifugation (1 min at 10 G) or stood for a few minutes before replating in hESF9a medium with 5 μM RI. Medium was changed daily.

### Embryoid bodies formation

*In vitro* differentiation was induced by the formation of embryoid bodies as described previously^14^. Undifferentiated hiPSCs were cultured by floating in DMEM-F12 medium supplemented with 20% KSR, 0.1 mM 2-mercaptoethanol, and MEM non-essential amino acids (Life Technologies). The floating embryoid bodies were then replated onto 1 mg/ml gelatin-coated dishes in DMEM with 10% FBS. The medium was changed every other day with the same floating culture solution.

### Karyotype analysis

Metaphase spreads were prepared from cells treated with colcemid (KaryoMAX Colcemid, Gibco 15212-012, final concentration of 40 ng/ml, overnight treatment) or metaphase arresting solution (Genial Genetic Solutions Ltd., Cheshire, UK). We performed a standard G-banding or multicolour fluorescence in situ hybridization (FISH) karyotypic analysis on at least 20 metaphase spreads for each population. For FISH karyotype analysis, 24XCyte Human Multicolor FISH Probe Kit (MetaSystems GmbH Altlussheim, Germany) were used.

### Cell detachment and dissociation assay

The dose response size and removal ratio of hPSCs cultured on divalent cations using 24-well plates were measured as follows. The cells cultured in ESF9a on fibronectin-coated dishes were detached and dissociated into cells clump using 0.2–0.5 U/ml dispase, and then were plated in ESF9a medium on 24-well plates coated with 2 μg/cm^2^ fibronectin (Wako) at 37°C for more than 1 hour. At 4 or 5 days after plating, the attached cells were stained with 1 μM calcein-AM (Dojindo, Kamimashiki, Kumamoto, Japan), a fluorescent living cell dye, for 20 min at 37°C, and imaged as the control state. Then the cells were rinsed once with PBS^−/−^, rinsed again with PBS containing various concentration of Ca^2+^ and Mg^2+^, incubated in the same PBS for a further 15 min at 37°C, and then triturated 5 times with a 1-ml micropipette tip. For enzymatic digestion experiments, dispase or trypsin was added after 12 min incubation in PBS and left for 3 min. The cells were then triturated 5 times with a 1-ml micropipette tip in the presence of 1 mg/ml DNase I (Roche, Basel, Switzerland), 250 μg/ml trypsin inhibitor (Life Technologies), and 1 mg/ml BSA (sigma), and then 10X volumes of PBS^ca/−^ were added before spinning down the cells. The detached cells were then transferred to another plate, and the remaining cells were imaged for green fluorescence to estimate detachment efficiency. The detached cell clumps were placed between cover slips and cell clump size was estimated based on fluorescent signal using Image J software (NIH, Bethesda, MD, USA). To estimate the cell clump sizes, randomly picked cell clumps for each condition in a test were analyzed with Excel software (Microsoft, Redmond, WA, USA). The cell clump size was converted from area (μm^2^) into the number of cells by using the area of single cells, which was estimated to be 240 ± 86 μm^2^ (mean ± SD, n = 107) in a separate experiment.

### Teratoma formation

Teratomas were generated in severe combined immunodeficient (SCID) mice from 201B7 hiPSCs grown under ESF-Fb-EzF conditions for more than 10 passages. The cells harvested by dispase were resuspended in DMEM supplemented with RI (10 μM). The cells from a confluent single well in a 6-well plate were injected into the thigh muscle of a SCID (C.B-17/lcr-scid/scidJcl) mouse (CLEA Japan, Tokyo, Japan). Nine weeks after injection, tumors were dissected, weighed, and then fixed with 10% formaldehyde Neutral Buffer Solution (Nacalai Tesque, Kyoto, Japan). Paraffin-embedded tissue was sectioned and stained with hematoxylin and eosin (HE). All animal experiments were conducted in accordance with the guidelines for animal experiments of the National Institute of Biomedical Innovation, Osaka, Japan.

### Alkaline phosphatase (ALP) staining, immunocytochemistry

The hPSCs were stained with an Alkaline Phosphatase Staining Kit II according to the manual (StemGen, Cambridge, MA, USA). Briefly, the cells were rinsed twice with PBS^+/+^ and fixed with Fix Solution from the kit at room temperature for 4 minutes. The fixed cells were rinsed with PBS containing with 0.05% (v/v) Tween20 and incubated in AP Substrate Solution at room temperature for 20 to 30 minutes. Then the cells were rinsed with PBS^+/+^ and photographed.

Immunocytochemistry was performed as described previously^14^,^17^. Briefly, hiPSCs were fixed in 4% formaldehyde with 0.5 mM MgCl_2_ and 0.5 mM CaCl_2_. Then the cells were permeabilized and blocked with PBS containing 0.1–0.2% Triton X-100, 10 mg/ml BSA, 0.5 mM MgCl_2_, and 0.5 mM CaCl_2_, and then reacted with primary antibodies in the solution. The primary antibody binding was visualized using secondary antibodies. Antibody information is listed in Supplementary Table 2. Nuclei were stained with 1 μM DAPI (Wako). Micrographs were taken using a BZ-8100 fluorescence microscope (Keyence, Osaka, Japan).

### Flow cytometry (FCM)

FCM analysis was performed as described previously^14^,^17^. All cells were removed from culture dishes using 0.02% (w/v) EDTA-4Na in PBS^−/−^ and then fixed in 4% formaldehyde. The fixed cells were permeabilized and blocked with PBS^−/−^ containing 0.1–0.2% Triton X-100 and 10 mg/ml BSA, and then reacted with primary antibodies in the solution. The primary antibody binding was visualized with secondary antibodies. Antibody information is listed in Supplementary Table 2. A cell sorter (JSAN, Bay Bioscience Co., Ltd., Hogo, Japan) was used for data acquisition.

### Apoptosis

An annexin V-FITC apoptosis detection Kit was used to detect cell surface phosphatidylserine (BioVision, Milpitas, CA, USA). Cells were floating cultured for four hours in ESF9a solution following detachment and dissociation. For FCM analysis, the cells were re-dissociated by incubating in 0.02% EDTA solution followed by trituration using a 1-mL pipette tip. The living cells were then stained with FITC conjugate annexin V (1:100) and 50 μg/ml propidium iodide in binding buffer. The living cells nuclear were stained with 1 μg/ml Hoechst 33342 (Dojindo, Osaka, Japan).

### Spot sheet formation

Silicone rubber masks made of polydimethylsiloxane (PDMS, Sylgard 184, 10:1 mix; Dow Corning) were perforated with 2-mm-diameter holes using a hole-punch. The bacterial culture dish (non-cell-attachment-treated dishes, Iwaki) with PDMS masking were treated for 60 seconds by air plasma to make hydrophilic spots (YHS-R, SAKIGAKE-Semiconductor Co., Ltd), then coated with 2 μm/cm^2^ fibronectin for more than 1 hour at 37°C to make fibronectin spots. After rinsing twice with PBS^−/−^, the PDMS mask was removed and the dish was sterilized under a UV lamp. hPSCs (253G1) cultured under the ESF-Fb-Dsp condition or hiPSC Tic-derived hepatic progenitors were dissociated in calcium- and magnesium-free solution, and then plated in ESF9a solution with 5 μg/ml RI or in CDM medium with 50 ng/ml FGF10 with RI.

Early differentiation was induced by 2 days cultivation in the ESF6 medium with 2 ng/ml recombinant human BMP4 (314-BP, R&D Systems, Inc, Minneapolis, MN, USA) as described previously^17^. Hepatic progenitors were differentiated based on the previously reported protocol^25^. Briefly, the hiPSC Tic line was passaged and grown for 2 days in CDM medium^26^. hiPSCs were then grown for 3 days in CDM/PVA medium^26^ with 100 ng/ml activin, 20 ng/ml basic FGF, 10 ng/ml BMP4, and 10 μM LY294003 (9901, Cell Signaling Technology, Beverly, MA, USA), followed by 3 days differentiation in CDM/PVA medium with 50 ng/ml recombinant human FGF10 (345-FG-025/CF, R&D Systems).

### Data analysis

Image analyses were performed with Image J software (NIH). Statistical analyses were performed with R software (http://www.r-project.org).

## Author Contributions

K.O. prepared all figures and supplementary figures. S.T. prepared figure 1, 2 and 4 and supplementary figure 1, 3, 4, 5 and 6. A.F., K.Y. and M.K.F. prepared Figure 3, 4 and Supplementary Figure 7. Y.I., Y.O. and M.A. prepared supplementary figure 6. Y.H., T.C., T.M. and M.A. prepared Supplementary Figure 2. K.O., Y.H. and M.K.F. wrote the manuscript text. All authors reviewed the manuscript.

## Supplementary Material

Supplementary Information Supplementary Infomation

## Figures and Tables

**Table 1:** Passaging protocols for hPSC culture

Passage Protocols				Culture Conditions		References
Divalent cations	Enzymes	Manipulation	Cell clump size	Serum replacement	Coat** & feeder	
Ca^2+^, Mg^2+^ *	Collagenase	Cutting, Scraping, Glass beads	Large	KSR,	MEF	1,27
Ca^2+^, Mg^2+^ *	Free	Cutting	Large	KSR	MEF	5
Ca^2+^ ***	Trypsin & Collagenase	Pipetting	Large	KSR	MEF	2,6,23
Free (EDTA)	Trypsin TrypLE	Pipetting	Single cells, Large	KSR	MEF	4,5,6,19,28
Free (EDTA)	Free (CDB#)	Pipetting	Single cells	KSR	MEF	5
Ca^2+^, Mg^2+^ *	Dispase, Collagenase TrypLE	Scraping, Pipetting	Large	Free	Mg, Gx, Lm, Vn, Fb, Cg, pVn, pBSP	8,14,15,20,29
Ca^2+^, Mg^2+^ *	Free	Cutting	Large	Free	Vn	12
Free (EDTA)	Free (CDB#)	Pipetting	Small	Free	Mg, HBP	8,30
Ca^2+^	Free	Pipetting	Large (Small)	Free	Fb	Present study

*DEME/F12 or ESF solution^16^ containing Ca^2+^, Mg^2+^.

**MEF: mouse embryonic fibroblast feeder cells on gelatin-coated dishes, Mg: Matrigel, Gx: geltrex, Lm: laminin, Vn: vitronectin, Cg: collagen, pVn: vitronectin-derived peptide, pBSP: bone sialoprotein-derived peptide, HBP: heparin-binding peptide.

***Dissociation solution named CTK containing CaCl_2_, trypsin, collagenase IV, and KSR^23^. KSR contains many divalent cations.

# CDB: Cell dissociation Buffer (Life Technologies).
