# Supplementary material for: Enzyme-free Passage of Human Pluripotent Stem Cells by Controlling Divalent Cations
Source: Sci Rep. 2014 Apr 11;4:4646. doi: 10.1038/srep04646 (PMC3983606; doi:10.1038/srep04646)
Supplement: Supplementary Information — Supplementary Infomation [file srep04646-s1.docx]

Supplementary Information

Enzyme-free Passage of Human Pluripotent Stem Cells by Controlling Divalent Cations

Kiyoshi Ohnuma^*1,2^, Ayaka Fujiki^3^, Kana Yanagihara^3^, Saoko Tachikawa^1^, Yohei Hayashi^2^, Yuzuru Ito^4^, Yasuko Onuma^4^, Techuan Chan^2^, Tatsuo Michiue^2^, Miho K Furue^3^, Makoto Asashima^2,4^

1 Top Runner Incubation Center for Academia-Industry Fusion, Nagaoka University of Technology, 1603-1 Kamitomioka, Nagaoka, Niigata 940-2188, Japan

2 Department of Life Sciences (Biology), Graduate School of Arts and Sciences, The University of Tokyo, 3-8-1 Komaba, Meguro, Tokyo 153-8902 Japan

3 Laboratory of Stem Cell Cultures, Department of Disease Bioresources Research, National Institute of Biomedical Innovation, 7-6-8, Saito-Asagi, Ibaraki, Osaka 567-0085, Japan

4 Research Center for Stem Cell Engineering, National Institute of Advanced Industrial Science and Technology (AIST), Tsukuba Central 4, 1-1-1 Higashi, Tsukuba, 5 Ibaraki 305-8562, Japan

*Correspondence should be addressed to:

Kiyoshi Ohnuma (kohnuma@vos.nagaokaut.ac.jp, Tel&Fax +81-258-47-9454)

# Supplementary Methods

### Culture of hiPSCs

The hESC H9[^1^](#_ENREF_1), KhES1, and KhES3[^2^](#_ENREF_2) lines, as well as hiPSC 253G1[^3^](#_ENREF_3), 201B7[^4^](#_ENREF_4), and Tic[^5^](#_ENREF_5) lines were maintained in a KSR-based medium containing DMEM-F12 medium (Life Technologies, Grand island, NY, USA) supplemented with 20% (v/v) KSR (Life Technologies), 0.1 mM 2-mercaptoethanol (Sigma, St. Louis, MO, USA), MEM non-essential amino acids (Life Technologies), and 5-10 ng/ml recombinant human basic FGF (Wako Pure Chemical Industries, Ltd., Ibaraki Osaka, Japan) on mitomycin C (Wako)-treated mouse embryonic fibroblast (MEF) feeder cells in 1 mg/ml gelatin-coated dishes. For subculturing, the cells were detached from the culture dish using CTK medium consisting of 0.25% trypsin, 0.1% collagenase IV, 20% KSR, and 1 mM CaCl_2_ in PBS, and then replated in hESF9a medium with 5 µM ROCK inhibitor (Y-27632; Wako) (KSR-MEF-CTK condition)[^2^](#_ENREF_2)^,^ [^4^](#_ENREF_4)^,^ [^6^](#_ENREF_6)^,^ [^7^](#_ENREF_7).

The hESC HUES8[^8^](#_ENREF_8) line was maintained by the same protocols except for the culture medium, which comprised DMEM-F12 medium (Life Technologies), 0.44% (v/v) human plasma protein fraction (Baxter International Inc., Deerfield, IL, USA), 10% (v/v) KSR (Life Technologies), 0.1 mM 2-mercaptoethanol (Sigma), MEM non-essential amino acids (Life Technologies), and 5-10 ng/ml recombinant human basic FGF (Wako)[^9^](#_ENREF_9). The MEFs were cultured in DMEM medium supplemented with 10% fetal calf serum, 1% penicillin, and 1% streptomycin.

The serum-free hESF9a medium comprises hESF-Gro medium (Cell Science & Technology Institute, Miyagi, Japan)[^10^](#_ENREF_10) supplemented with 10 µg/ml bovine pancreas insulin (Sigma I-5500), 5 µg/ml human apotransferrin (Sigma T-1147), 10 µM 2-mercaptoethanol (Sigma M-7522), 10 µM ethanolamine (Sigma E-0135), 20 nM sodium selenite (Sigma S-9133), 4.7 µg/ml of oleic acid conjugated with 0.5 mg/ml of fraction V fatty acid-free bovine serum albumin (Sigma O-3008), 100 ng/ml L-ascorbic acid-2-phosphate (Wako, Osaka, Japan, 013-196411), 100 ng/ml heparin sodium salt from porcine intestinal mucosa (Sigma H-3149), 10 ng/ml basic FGF (Wako), and 2 ng/ml human recombinant activin A (338-AC R&D Systems, Minneapolis, MN, USA) [^7^](#_ENREF_7)^,^ [^11^](#_ENREF_11)^,^ [^12^](#_ENREF_12).

The experiments using hESCs were performed following the Guidelines for utilization of hESCs of the Ministry of Education, Culture, Sports, Science and Technology of Japan with the approval by each institutional research ethics committee.

# Supplementary Figures

### Supplementary Figure 1: Dose-response effects of Ca^2+^ and Mg^2+^ on cell detachment ratio and on detached cell clump size from fibronectin-coated dishes

The hiPSC 201B7 line was used to obtain these results, with the same experimental protocols as for **Fig. 1** (hiPSC 253G1 line).

**ab**: Fluorescent micrograph of hiPSCs stained with calcein-AM on a fibronectin-coated 24-well plate. After 15 minutes of incubation in PBS containing 0, 10, 100, or 1000 µM Ca^2+^ and Mg^2+^, the cells were triturated 5 times with a 1-ml pipette tip and rinsed. The cells remaining on the well surface are shown (**a**). Higher concentrations of Mg^2+^ and Ca^2+^ retained more cells attached to the plate, with a stronger dependence on Mg^2+^concentration. **b**: The dissociated cells were transferred to a cover slip for examination. The dissociated cells formed larger clumps at higher concentrations of Ca^2+^.


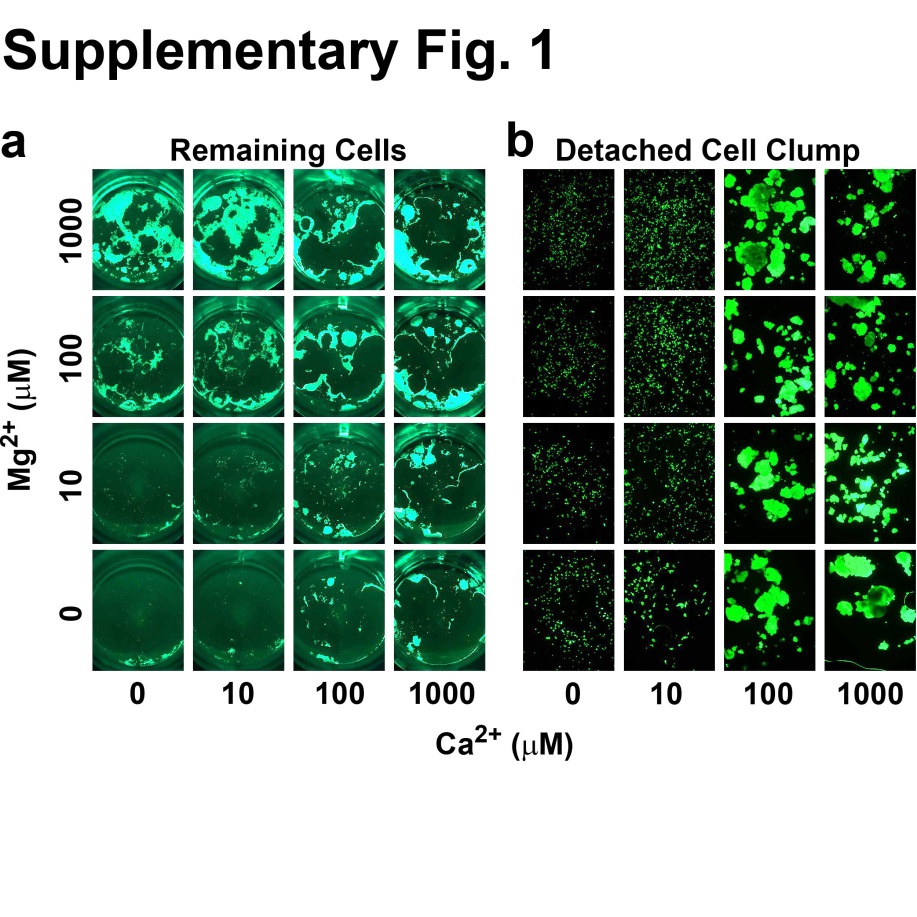


### Supplementary Figure 2. hESCs detached from and reattached on fibronectin-coated dishes in solution with Ca^2+^ and without Mg^2+^

**a-g**: HUES8 (**a-d**) and H9 (**e-g**) hESCs colonies cultured in ESF9a on fibronectin-coated dishes (**ae**). The hESC colonies began to detach from the dish after 15 minutes incubation in PBS^-/Ca^ (**bf**), and most undifferentiated colonies were detached from the dishes after trituration (**c**). Spontaneously differentiated cells surrounding the undifferentiated cell colonies were not detached from the dishes (**c**). The detached cells formed large colonies (**dg**). Scale bars are 1 mm.

**h-m**: One day after reseeding, the detached HUES8 (**hij**) and H9 cells (**klm**) were reattached to a fibronectin-coated dish, again in ESF9a medium. Phase-contrast micrograph (**h**), APL staining (**k**). **ijlm**: Immunocytochemistry for SSEA4 (green) and oct3/4(red). **jm**: Magnified images of the boxed areas in **il**, respectively. Nuclei were stained with DAPI (blue). Scale bars are 10 mm (**k**), 1 mm (**hil**), and 100 μm (**jm**).


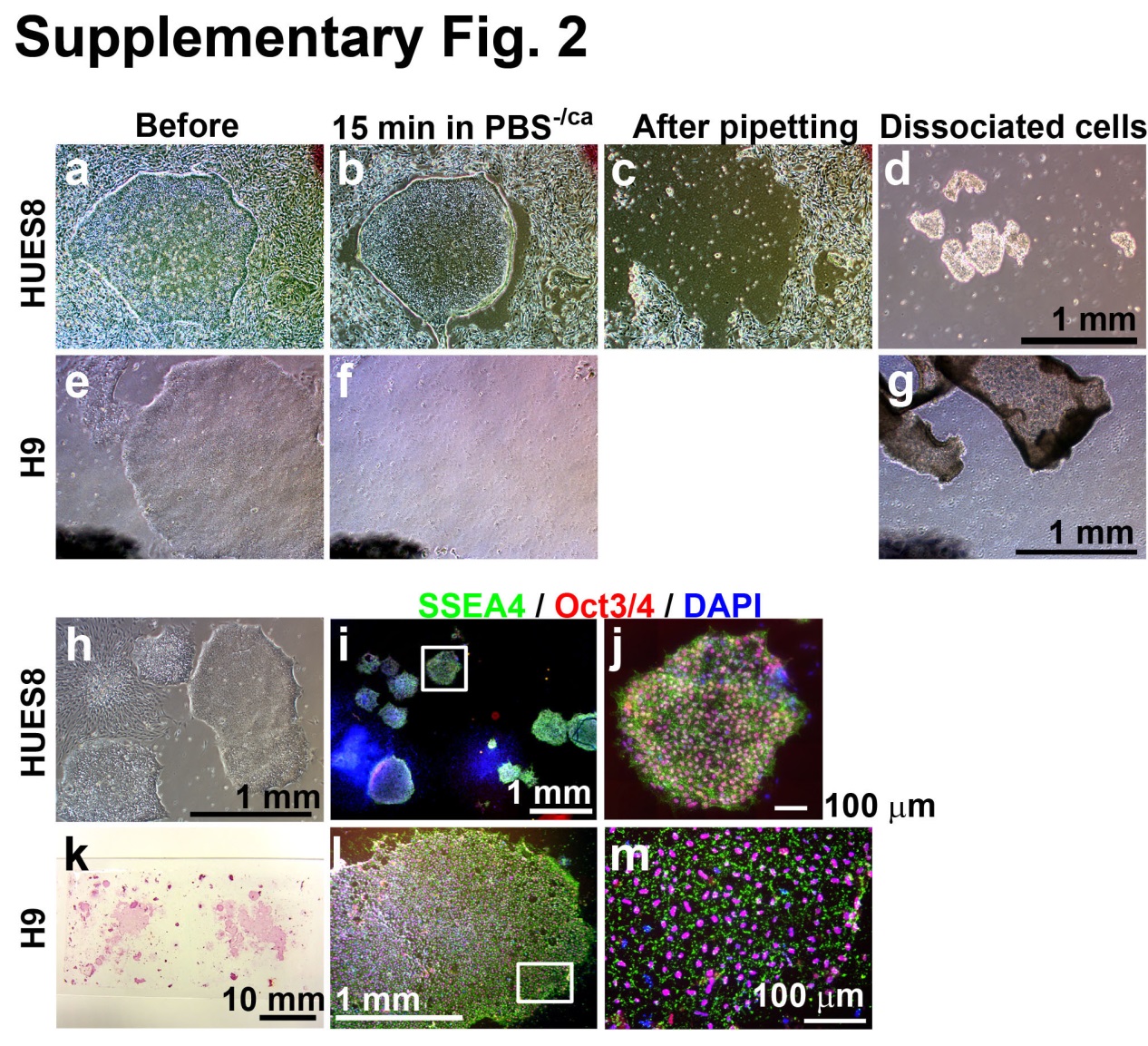


### Supplementary Figure 3: Cell detachment from vitronectin- or laminin-coated wells by incubation in PBS with and without Ca^2+^ and Mg^2+^

253G1 and 201B7 hPSCs cultured in ESF9a on fibronectin-coated dishes were detached and dissociated into single cells using 0.02% (v/v) ethylenediaminetetraacetic acid (EDTA)-PBS. These cells were then seeded at sub-confluent density (1 x 10^5^ cells per cm^2^) in ESF9a medium with 5 μM ROCK inhibitor onto 96-well microplates (TPP) coated with 0.5 μg/cm^2^ vitronectin (Vn) and 5 μg/cm^2^ laminin (Lm) at 37°C for at least 1 hour. On the next day, the attached cells were rinsed once with PBS^-/-^, rinsed again with PBS^-/-^ (blue), PBS^Mg/-^ (red), PBS^-/Ca^ (green), or PBS^Mg/Ca^ (purple), and then incubated in the same PBS for 15 min at 37°C. Then the cells were triturated three times using a micropipette tip to detach the hPSCs. The numbers of remaining, non-detached cells were estimated using crystal violet, as described previously^1^. Briefly, after rinsing twice with PBS containing 0.5 mM MgCl_2_ and 0.5 mM CaCl_2_ (PBS^+/+^), the remaining cells were fixed and stained for 30 minutes with 0.4% crystal violet (Sigma-Aldrich) in methanol at room temperature. After the plate was washed and dried, a dye-eluting solution comprising 1% acetic acid and 30% ethanol in water was added to the wells. The absorbance at 595 nm was measured with a microplate reader (model 680; Bio-Rad, Hercules, CA), to indicate the concentration of crystal violet eluted from the remaining cells.

Each graph shows the percentage of absorbance at 595 nm (remaining cells) relative to the absorbance without incubation in PBS or trituration. Two-way ANOVA found significant differences in cell number between different Ca^2+^ concentrations in all conditions. No interactions were found between Ca^2+^ and Mg^2+^ except for 253G1 on vitronectin, suggesting the effects were also dependent on Ca^2+^ in these conditions (mean ± SE, n = 3, *P*-values are shown on the graph with significant values where *P* < 0.05 shown in red type. Ca and Mg: main effect on Ca^2+^ and Mg^2+^ concentration, respectively. Int: interaction between Ca^2+^ and Mg^2+^). These results suggested that the hiPSCs on vitronectin and laminin could be detached in the buffer without Ca^2+^, but that the extent of detachment could differ with cell type.


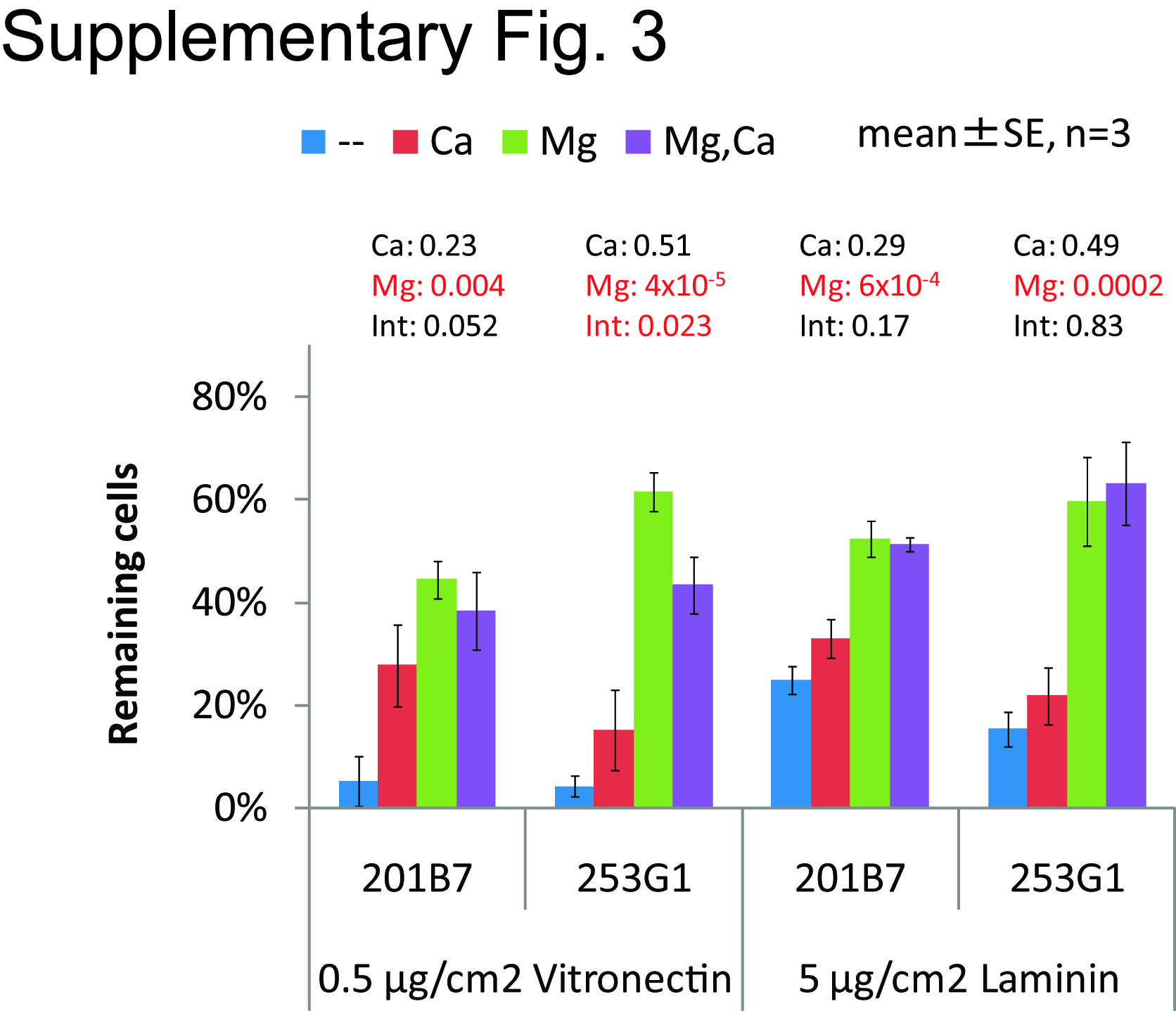


### Supplementary Figure 4: Effects of dissociation without divalent cation and with dispase

The hiPSC 201B7 line was used except for **c-right** (Tic), with the same experimental protocols as for **Fig. 2** (hiPSC 253G1 line).

**a-c:** Comparison between the hiPSCs detached and dissociated by PBS^-/-^ (-/-) and those by PBS^ca/-^ (Ca).

**ab:** Microphotograph (**a**) and flow cytometrical (FCM) analysis (**b**) of apoptosis marker annexin V-FITC after four hours in floating culture (RI: 5 μM ROCK inhibitor was added) following detachment and dissociation (201B7). The cells were stained by annexin V-FITC (green), propidium iodide (red: late apoptosis or necrosis marker). Bright field micrographs are shown as blue images. Scale bars are 100 μm. (* *P* = 0.05, n.s.: not significant, n = 6, mean ± SE. Because **b, f, Supplementary Fig. 5g** data were taken at the same time, the PBS^ca/-^ data is identical. Thus, Holm’s multiple comparison tests were performed). **c:** The number of cells after 3 days culture in ESF9a on fibronectin-coated 10-cm dishes (the initial number of cells was 1.2 x 10^5^ cells/10-cm dish, mean ± SE, t-test)(**left:** 201B7, n = 13, **right:** Tic, n = 4).

**d-g:** Effects of dispase in PBS^ca/-^ for detaching and dissociating cells. Dispase was added after 12 minutes incubation in PBS and left for a further 3 minutes. In all experiments, the cells were triturated 5 times in the presence of 1 mg/mL DNase I (Roche, Basel, Switzerland), 250 μg/mL trypsin inhibitor (Life Technologies), and 1 mg/mL BSA (Sigma), and then 10x volumes of PBS^ca/-^ were added before spin-down. **d:** Mean cell clump size detached from the plates using PBS^ca/-^ and 1 U/ml dispase in PBS^ca/-^. (* *P* = 0.01, ns: not significant, n = 4 experiments x 200 cells, mean ± SE. Because **d** and **Supplementary Fig. 5e** data were taken at the same time, the PBS^ca/-^ data is identical. Thus, Holm’s multiple comparison tests were performed). **ef:** Microphotograph (**e**) and flow cytometrical analysis (**f**) of annexin V-FITC after four hours in floating culture (RI: 5 μM ROCK inhibitor was added) following detachment and dissociation by PBS^ca/-^ or 1 U/ml dispase in PBS^ca/-^. The cells were stained by annexin V-FITC (green), propidium iodide (red). Bright field micrographs are shown as blue images. Scale bars are 100 μm. (Please see **b** for statistical analysis) **g**: Reattachment efficiency. The cells were digested with 0-2 U/ml dispase in PBS^ca/-^ and then plated in 24-well plates with ESF9a medium including 5 μM RI. Cell numbers were estimated using calcein-AM 1 day after plating and normalized against the PBS^ca/-^ results (0 U/ml dispase). The mean value at 1 U/ml dispase was smaller than 1 (*P* < 0.001, n = 7, t-test). Pearson's correlation coefficient = -0.739, *P* = 2.2 x 10^-8^ t-test, 7 independent experiments.


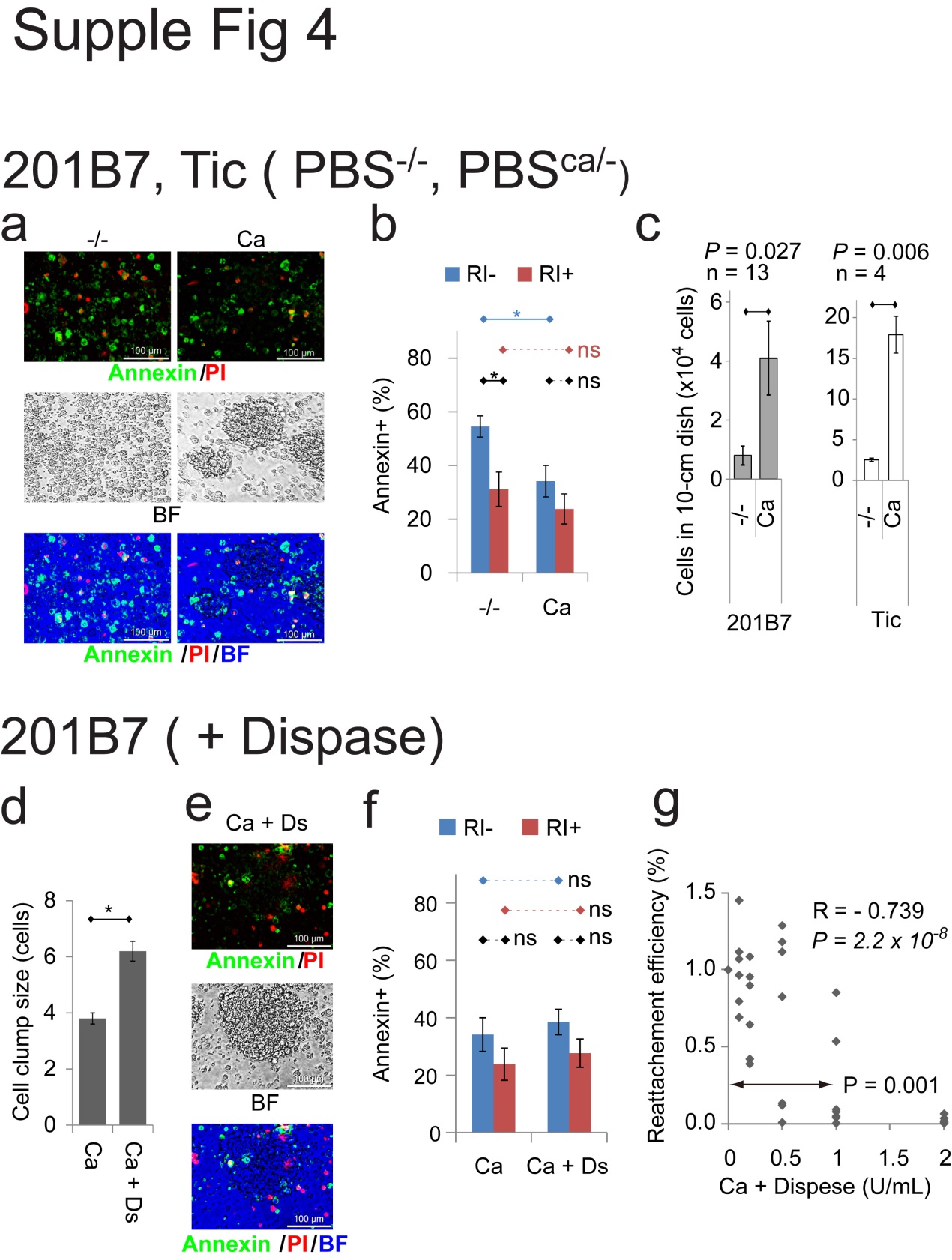


### Supplementary Figure 5: Effects of trypsin

Effects of dispase in PBS^ca/-^ for detaching and dissociating hiPSC 253G1 (**a-d**) and 201B7 lines (**e-h**). The experimental protocols were the same as those used for **Fig. 2d-g** except for using trypsin instead of dispase (**a** and **e** include dispase data for comparison).

Trypsin (or dispase) was added after 12 minutes incubation in PBS and left for a further 3 minutes. In all experiments, the cells were triturated 5 times in the presence of 1 mg/ml DNase I (Roche), 250 μg/ml trypsin inhibitor (Life Technologies), and 1 mg/ml BSA (Sigma), and then 10 x volumes of PBS^ca/-^ were added before spin-down.

**ae:** Mean cell clump size detached and dissociated from the plates using PBS^-/-^ (**-/-**) or PBS^ca/-^ (ca) with 0.25% trypsin (Tr) or 1 U/ml dispase (Ds). (* *P* = 0.01, ns: not significant, n = 4 experiments x 200 cells, mean ± SE. Holm’s multiple comparison tests. **a** (253G1) and **e** (201B7) include the original **Fig. 2d** and **Supplementary Fig 4d**, respectively. These data were acquired at the same time.

**bcfg:** Microphotograph (**bf**) and flow cytometrical analysis (**cg**) of annexin V-FITC after four hours in floating culture (RI: 5 μM ROCK inhibitor was added) following detachment and dissociation by PBS^ca/-^ or 0.25% trypsin in PBS^ca/-^. The cells were stained by annexin V-FITC (green), propidium iodide (red), and live cell nuclear stain, Hoechst 33342 (blue, **b**). Bright field micrographs are shown as blue images (**f**). Scale bars are 100 μm. (* *P* = 0.05, ns: not significant, n = 6, mean ± SE. Because 253G1 (**c** and **Fig. 2bf**) and 201B7 data (**g** and **Supplementary Fig. 4bf)** were taken at the same time, each set of PBS^ca/-^ data is identical. Thus, Holm’s multiple comparison tests were performed).

**dh**: Reattachment efficiency. The cells were digested with 0-0.5% trypsin in PBS^ca/-^ and then plated in 24-well plates with ESF9a medium including 5 μM RI. Cell numbers were estimated using calcein-AM 1 day after plating and normalized against the PBS^ca/-^ results (0% trypsin). **d:** The mean value at 0.25% trypsin was smaller than 1. (*P* < 0.01, n = 4, t-test). Pearson's correlation coefficient = -0.839, (*P* = 3.0 x 10^-7^).

**h:** The mean value at 0.25% trypsin was smaller than 1. (*P* < 1 x 10^-5^, n = 5, t-test). Pearson's correlation coefficient = -0.742, (*P* = 3.4 x 10^-5^).


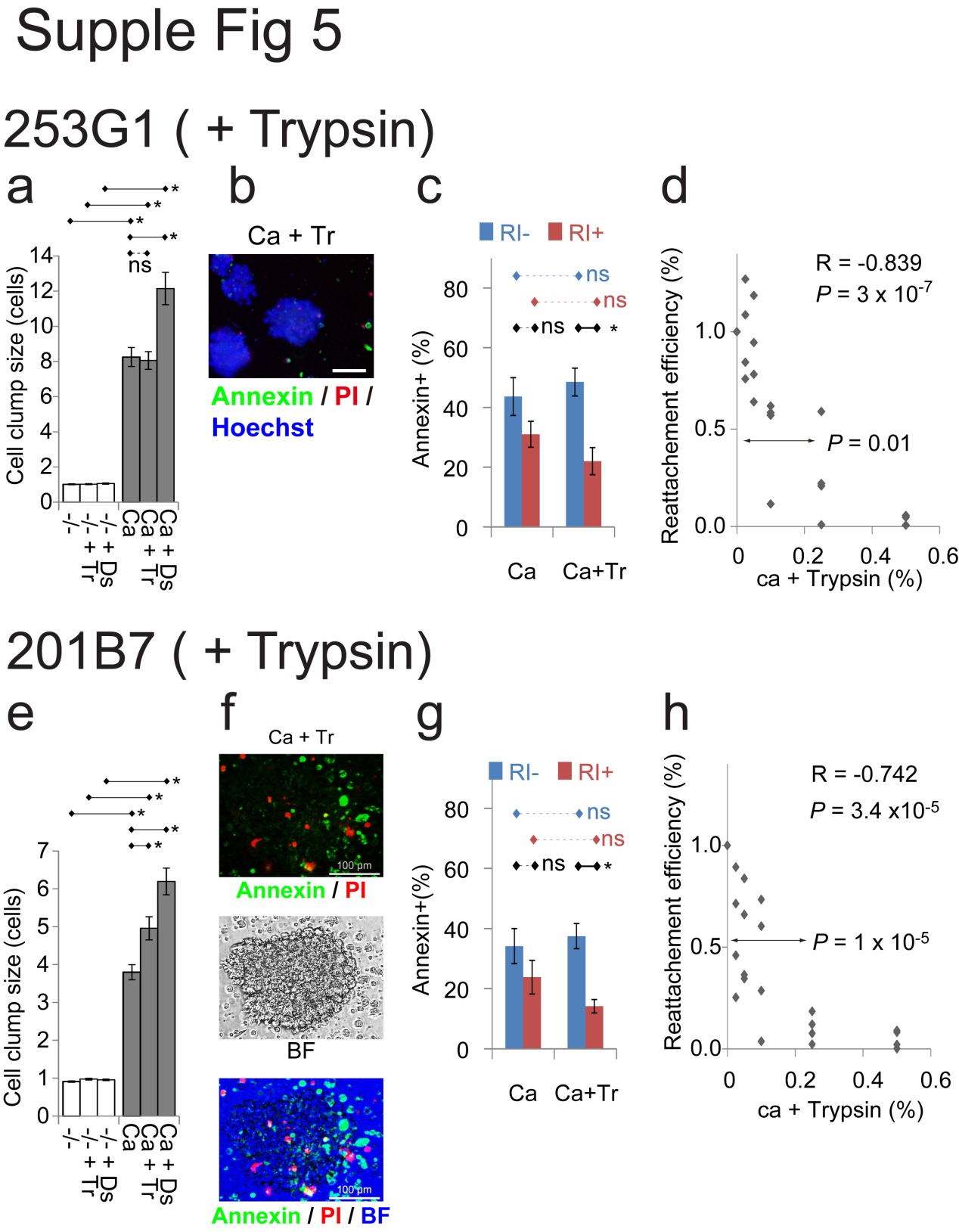


### Supplementary Figure 6: Passage solution with Ca^2+^ and without Mg^2+^ supports long-term culture of hPSC 253G1 and Tic lines

The hiPSCs 253G1 (**a-i**) and 201B7 (**j-r)** were cultured.

The hiPSC 253G1 line (**a-d, i**) and the 201B7 line (**j-m, r**) were cultured for more than 10 passages under the ESF-Fb-EzF condition. **aj:** Expression of self-renewal marker, ALP. **bk:** Expression of self-renewal markers Oct3/4 (red) and SSEA4 (Green). The nuclei were stained with DAPI (blue). The right panels are magnified images of the boxed area in the left panels. **cl:** FCM analysis for SSEA4 and oct3/4 (solid line). Dotted line: without primary antibody (negative control). **dm:** Immunohistochemistry of *in vitro* differentiated cells using embryoid body formation. Expression of the ectoderm marker GFAP, mesoderm markers Nkx2.5 and vimentin, and endoderm marker  feto protein in the differentiated hiPSC lines after long-term culture. Nuclei were stained with DAPI (blue).

**e-i, n-r**: Culture history trees (**en**) and karyotypes (**f-i, o-r**). 253G1 and 201B7 cells were obtained from the Cell Bank at passage 23 and 17, respectively (**CB**). Numbers indicate the passage number. Karyotypes of the 253G1 (**f-i**) and 201B7 (**o-r**) hiPSCs with the culture history represented on the left. **fgop**: Early (**fo**) and late (**gp**) passage-number cells under KSR-MEF-CTK (**K**) condition. **hiqr**: Cells cultured for 10 passages under ESF-Fb-Dsp (**SF**) condition (**hq**) and ESF-Fb-EzF (**EzF**) condition (**ir**). **i** and **r** corresponds to **a-d** and **j-m**, respectively. (**I**) Intermediate state: the cells were passaged in CTK medium and cultured in ESF9a medium on fibronectin-coated dishes. Arrows represent the highly probable point in the history when the karyotype change might have occurred.


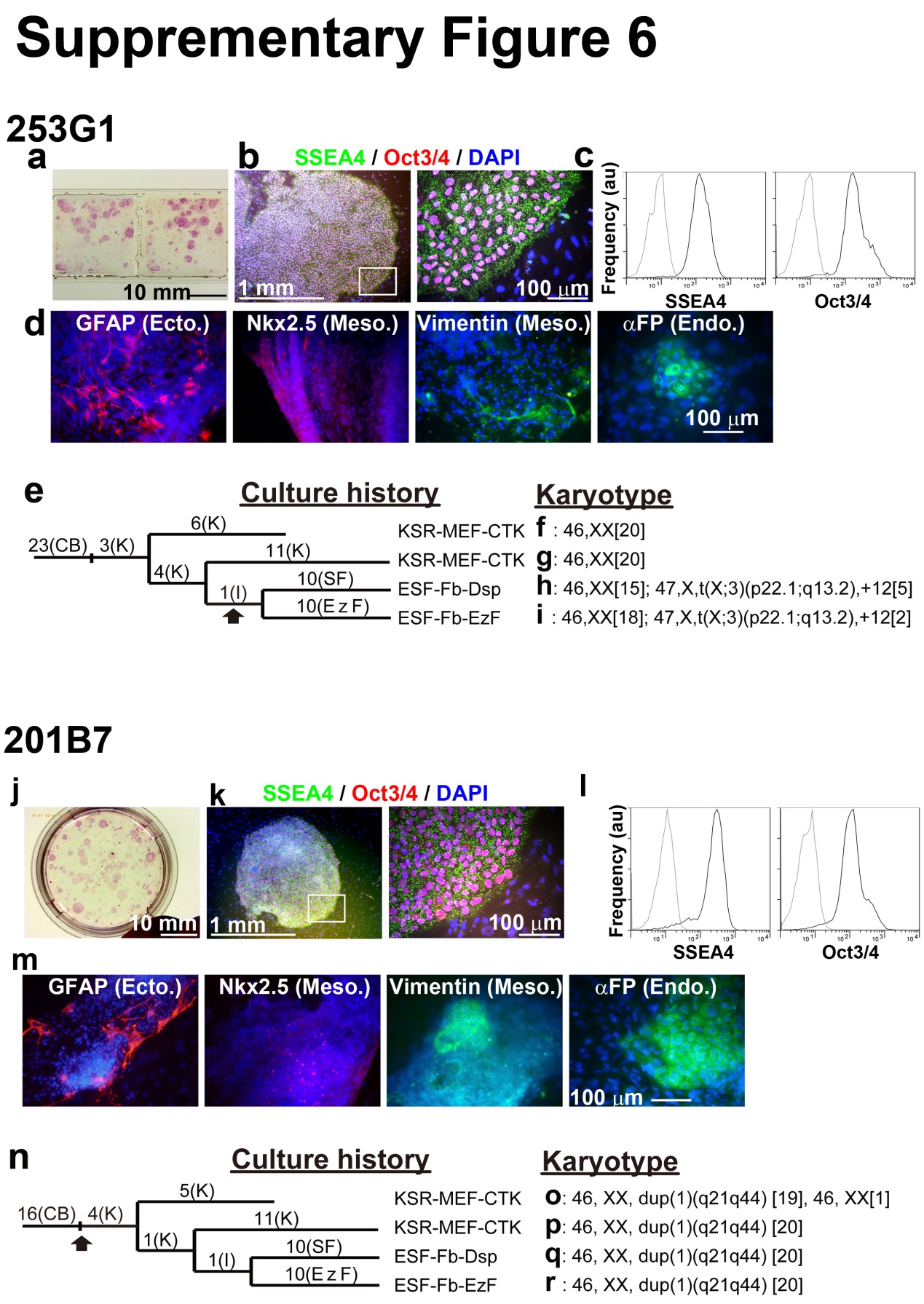


### Supplementary Figure 7: Passage solution with Ca^2+^ and without Mg^2+^ supports long-term culture of hPSC Tic line

The hiPSCs Tic line was cultured for 20 passages in the enzyme-free passage solution under serum- and feeder-free culture conditions (ESF-Fb-EzF condition). **a:** Phase contrast microscopy showed that the cells formed normal hiPSC colonies, which were tightly packed, and flat colonies consisting of cells with large nuclei and scant cytoplasm[^1^](#_ENREF_1)^,^ [^4^](#_ENREF_4). **b-f:** Immunocytochemistry showed that the cells expressed self-renewal markers, Nanog (**b:** red), Oct3/4 (**c:** red), SSEA4 (**c:** red) and Tra 1-60 (**e:** red), but not an early differentiation marker, SSEA1 (**f:** red). The nuclei were stained with DAPI (blue). Scale bars are 100µm (**a**) and 200 µm (**b-f**). **g:** FISH analysis showed a normal karyotype (46XY).


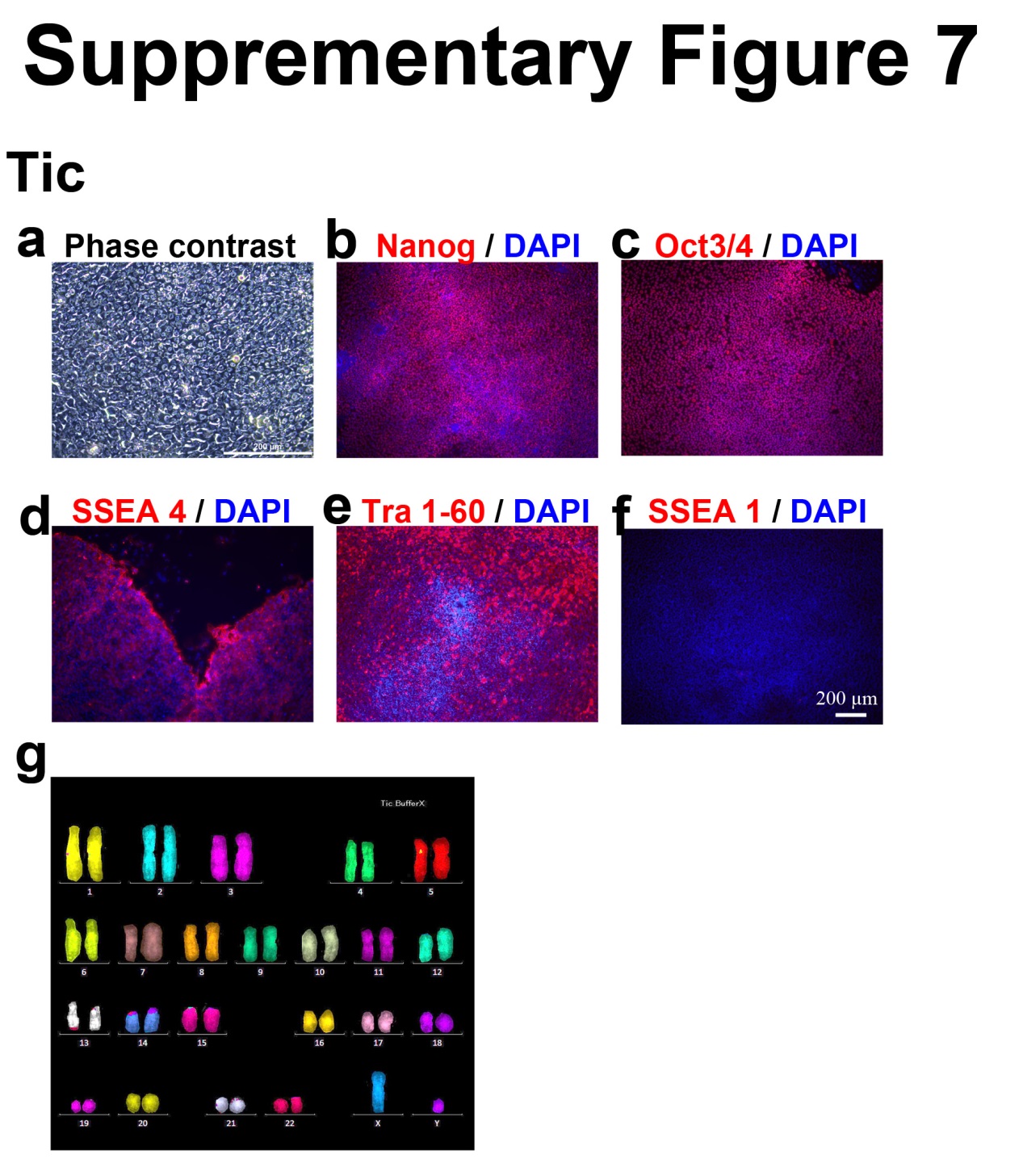


### Supplementary Table 1: Divalent cation-free DMEM-F12 medium (Wako, custom-made)

### Supplementary Table 2. Antibodies

| Markers | Primary antibody | Secondary antibody |
| --- | --- | --- |
| Oct3/4 | anti-human Oct3/4 (H-134)  rabbit polyclonal IgG  sc-9081, SantaCruz^1^  Dilution 1/500 | goat anti-rabbit IgG(H+L)  Alexa Fluor 546  A11035, Life Technologies^6^  Dilution 1/2000 |
| SSEA4 | anti-human SSEA4 (MC-813-70)  mouse monoclonal IgG3  MAB4304, Chemicon (Milipore^2^)  Dilution 1/1000 | goat anti-mouse IgG3 (γ3)  Alexa Fluor 488  A21151, Life Technologies^6^  Dilution 1/500 |
| TRA 1-60 | anti-human TRA-1-60 (TRA-1-60)  mouse monoclonal IgM  Sc-21705, SantaCruz ^1^  Dilution 1/100 | goat anti-mouse IgM (µ chain)  Alexa Fluor 488  A21042, Life Technology^6^  Dilution 1/500 |
| SSEA1 | Anti-human SSEA-1(480)  mouse monoclonal IgM  sc-21702, SantaCruz^1^  Dilution 1/100 | goat anti-mouse IgM (µ chain)  Alexa Fluor 488  A21042, Life Technology^6^  Dilution 1/500 |
| GFAP | anti-glial fibrillary acidic protein  rabbit polyclonal IgG  Z0334, DAKO^3^  Dilution 1/500 | goat anti-rabbit IgG (H+L)  Alexa Fluor 546  A11035, Life Technologies^6^  Dilution 1/2000 |
| Vimentin | anti-vimentin antibody (S-20)  goat polyclonal IgG  sc-7558, SantaCruz^1^  Dilution 1/200 | rabbit anti-goat IgG  Alexa Fluor 488  A21222, Life Technologies^6^  Dilution 1/500 |
| Nkx2.5 | anti-Nkx2.5 antibody (H-114)  rabbit polyclonal IgG  SC-14033, SantaCruz^1^  Dilution 1/200 | goat anti-rabbit IgG(H+L)  Alexa Fluor 546  A11035, Life Technologies^6^  Dilution 1/2000 |
| α-FP  (Suppl. Fig. 6) | anti-α-fetoprotein antibody (Clone  189502)  mouse monoclonal IgG1  MAB1368, R&D Systems^4^  Dilution 1/ | goat anti-mouse IgG1  Alexa Fluor 488  A21121, Life Technologies^6^  Dilution 1/500 |
| α-FP  (Fig. 4) | anti-human-alpha-1-fetoprotein  rabbit polyclonal IgG  A0008, Dako^3^  Dilution 1/200 | goat anti-rabbit IgG (H+L)  Alexa Fluor 488  A21206, Life Technologies^6^  Dilution 1/500 |
| epCAM | anti-EpCAM antibody (HEA125)  mouse monoclonal IgG1  ab46714, abcam^5^  Dilution 1/10 | goat anti-mouse IgG1 (γ1)  Alexa Fluor 546  A21123, Life Technologies^6^ Dilution 1/2000 |
| iCAM | anti-human ICAM-1/CD54 (Clone BBIG-I1)  mouse monoclonal IgG1  BBA4, R&D Systems^4^  Dilution 1/200 | goat anti-mouse IgG1 (γ1)  Alexa Fluor 546  A21123, Life Technologies^6^  Dilution 1/2000 |

^1^ SantaCruz Biotechnology, Inc., CA, USA;

^2^ Millipore, Billerica, MA, USA;

^3^ Dako Denmark A/S, Inc., Glostrup, Denmark

^4^ R&D Systems, Minneapolis, USA;

^5^ Abcam, Cambridge, UK;

^6^ Life Technologies Inc., Carlsbad, California, USA;

### References

1. Thomson, J.A. et al. Embryonic stem cell lines derived from human blastocysts. *Science* **282**, 1145-1147 (1998).

2. Suemori, H. et al. Efficient establishment of human embryonic stem cell lines and long-term maintenance with stable karyotype by enzymatic bulk passage. *Biochem Biophys Res Commun* **345**, 926-932 (2006).

3. Nakagawa, M. et al. Generation of induced pluripotent stem cells without Myc from mouse and human fibroblasts. *Nature biotechnology* **26**, 101-106 (2008).

4. Takahashi, K. et al. Induction of pluripotent stem cells from adult human fibroblasts by defined factors. *Cell* **131**, 861-872 (2007).

5. Nishino, K. et al. DNA Methylation Dynamics in Human Induced Pluripotent Stem Cells over Time. *PLoS Genetics* **7**, e1002085 (2011).

6. Watanabe, K. et al. A ROCK inhibitor permits survival of dissociated human embryonic stem cells. *Nat Biotechnol* **25**, 681-686 (2007).

7. Hayashi, Y. et al. Reduction of N-Glycolylneuraminic Acid in Human Induced Pluripotent Stem Cells Generated or Cultured under Feeder- and Serum-Free Defined Conditions. *PLoS ONE* **5**, e14099 (2010).

8. Cowan, C.A. et al. Derivation of embryonic stem-cell lines from human blastocysts. *N Engl J Med* **350**, 1353-1356 (2004).

9. Chen, S. et al. A small molecule that directs differentiation of human ESCs into the pancreatic lineage. *Nat Chem Biol* **5**, 258-265 (2009).

10. Furue, M.K. et al. Heparin promotes the growth of human embryonic stem cells in a defined serum-free medium. *Proc Natl Acad Sci U S A* **105**, 13409-13414 (2008).

11. Yoshimitsu, R. Microfluidic Perfusion Culture of Human Induced Pluripotent Stem Cells under Fully Defined Culture Conditions. *Biotechnol Bioeng* (In press).

12. Na, J., Furue, M.K. & Andrews, P.W. Inhibition of ERK1/2 prevents neural and mesendodermal differentiation and promotes human embryonic stem cell self-renewal. *Stem cell res* **5**, 157-169 (2010).
